# A Rare Case of Osteomyelitis Presenting as Cyst-Like Lesion in the Coronoid Process: A Diagnostic Challenge

**DOI:** 10.7759/cureus.18616

**Published:** 2021-10-08

**Authors:** Rajashri Rajendiran, Madhulaxmi Marimuthu, Abdul Wahab, Geetha Sridharan

**Affiliations:** 1 Oral and Maxillofacial Surgery, Saveetha Dental College and Hospitals, Chennai, IND

**Keywords:** ct (computed tomography) imaging, oro-cutaneous fistula, anemia, cyst in relation to coronoid process, chronic osteomyelitis

## Abstract

Cyst-like lesion in the coronoid process of the mandible is a challenging diagnosis to make, as it may present with a range of non-specific symptoms. A middle-aged woman reported a one-year history of non-bloody, pus discharge from the right angle of the mandible. There was a history of prior surgery comprising teeth removal two years ago for a painful swelling on the right side of her face, following which her symptoms regressed but in the due course, she developed a chronic sinus with draining abscess. Radiographic findings, in combination with clinical symptoms, are critical in the diagnosis and evaluation of cysts and cyst-like lesions of the jaws. The orthopantomograph (OPG) revealed a cyst-like lesion in the coronoid process of the mandible with an extra-oral sinus tract leading to the epicenter of the cyst-like radiolucency, and so this, in combination with the patient’s atypical symptoms, presented a diagnostic challenge. This case report explores the events which led to the diagnosis of osteomyelitis and shows several unique learning points.

## Introduction

The presentation of a cyst-like lesion in the coronoid process of the mandible is rare and not commonly associated with an extra-oral sinus tract. Diagnosis in such cases is challenging for clinicians. Radiographic findings, in combination with clinical symptoms, are critical in the diagnosis and evaluation of cysts and cyst-like lesions of the jaws. Making a differential diagnosis based on the radiographic studies can negatively affect the treatment planning process because mandibular lesions may arise from both odontogenic and nonodontogenic origins. Since both odontogenic and nonodontogenic lesions mimic each other with similar radiological appearances, diagnosis may become challenging [1]. This article will review the importance of imaging of the cyst-like lesion in the coronoid process of the mandible in establishing the diagnosis that was subsequently confirmed by histopathology as osteomyelitis.

## Case presentation

**Figure 1:**
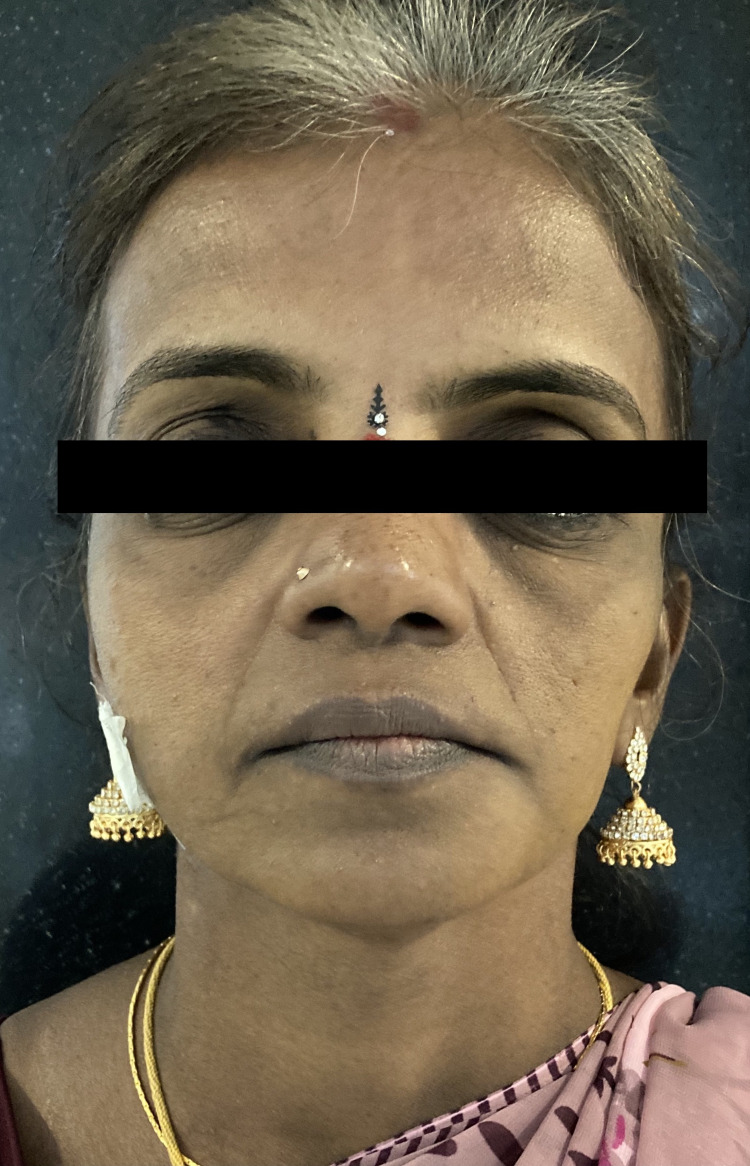
Pre-operative extra-oral frontal picture.

A 45-year-old female reported to the outpatient clinic, for evaluation of an extra-oral cutaneous fistula on the right angle of mandible draining pus for the past 18 months (Figure 1).

The patient gave a history of undergoing surgical procedure involving extraction of lower right back teeth for a painful swelling she had developed on the right side of her face. Her symptoms improved after the procedure until she developed pus discharge from her right cheek a few weeks after the procedure. She gave no history of pain and was completely asymptomatic. Her medical history revealed anemia.

**Figure 2:**
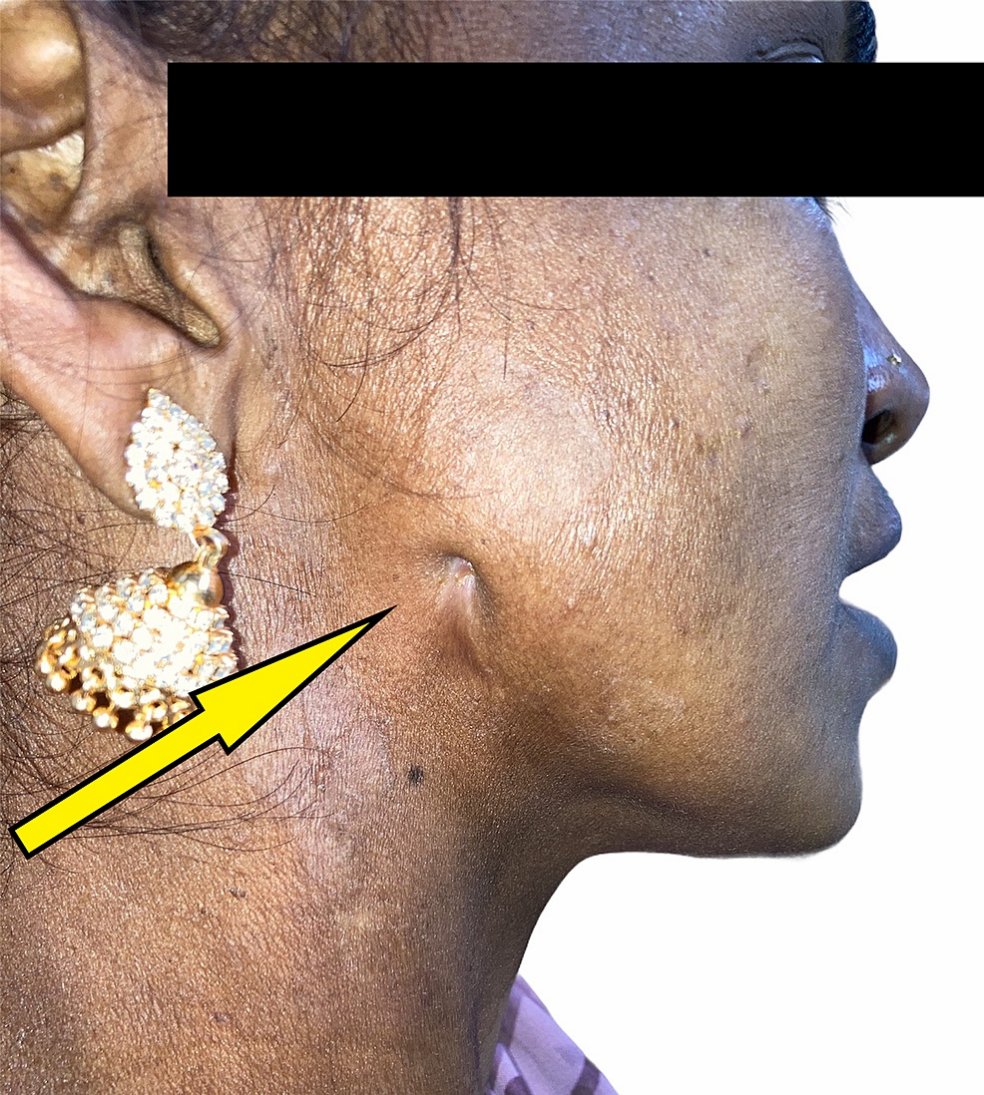
Pre-operative extra-oral profile showing oro-cutaneous fistula.

On initial physical examination, there was an extra oral cutaneous fistula measuring 0.4 mm x 0.5 mm on the right cheek. The skin over the opening was similar in color, texture, and consistency to the surrounding skin and showed no signs of inflammation (Figure 2).

**Figure 3:**
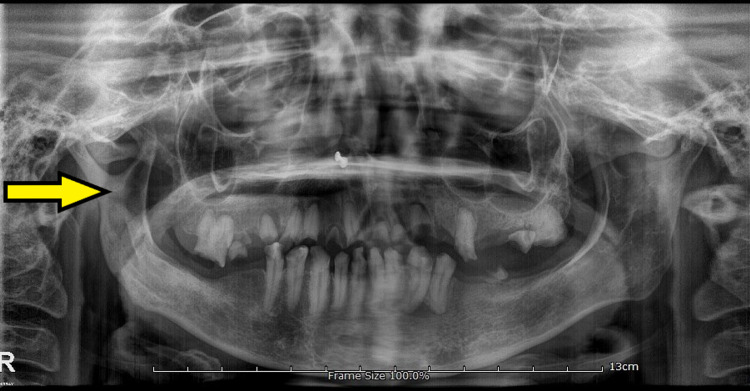
Pre-operative OPG showing a well-defined radiolucency measuring 16 mm x 11 mm in the right coronoid process along with multiple grossly decayed teeth in maxilla and mandible. OPG, orthopantomograph

The patient had an asymmetry of the face owing to a puffed cheek on the right side. Orthopantomograph (OPG) revealed a well-defined radiolucency in the right coronoid process along with multiple grossly decayed teeth (Figure 3).

**Figure 4:**
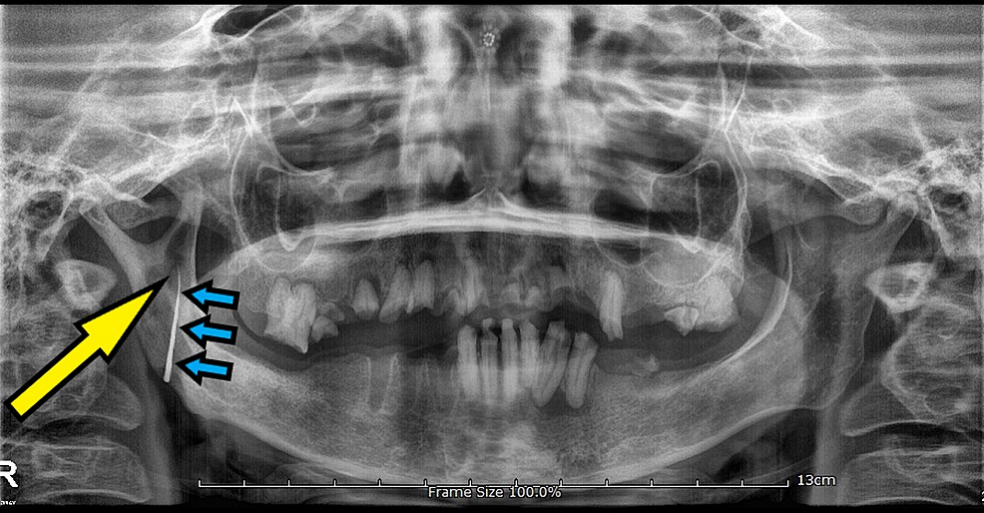
Pre-operative OPG showing cyst-like lesion in the right coronoid process (pointed by yellow arrow). Linear radiopacity is the tracer gutta-percha point passed through the extra-oral cutaneous tract (pointed by blue arrows). The gutta percha point is seen abutting the cyst-like lesion in the coronoid process. OPG, orthopantomograph

The cutaneous fistula did not open into the oral mucosal lining and hence a gutta percha point was used to trace the fistulous tract and an OPG was repeated. The tracer gutta percha point measuring 3 cm was abutting the right hemi-mandibular osseous defect (Figure 4).

**Figure 5:**
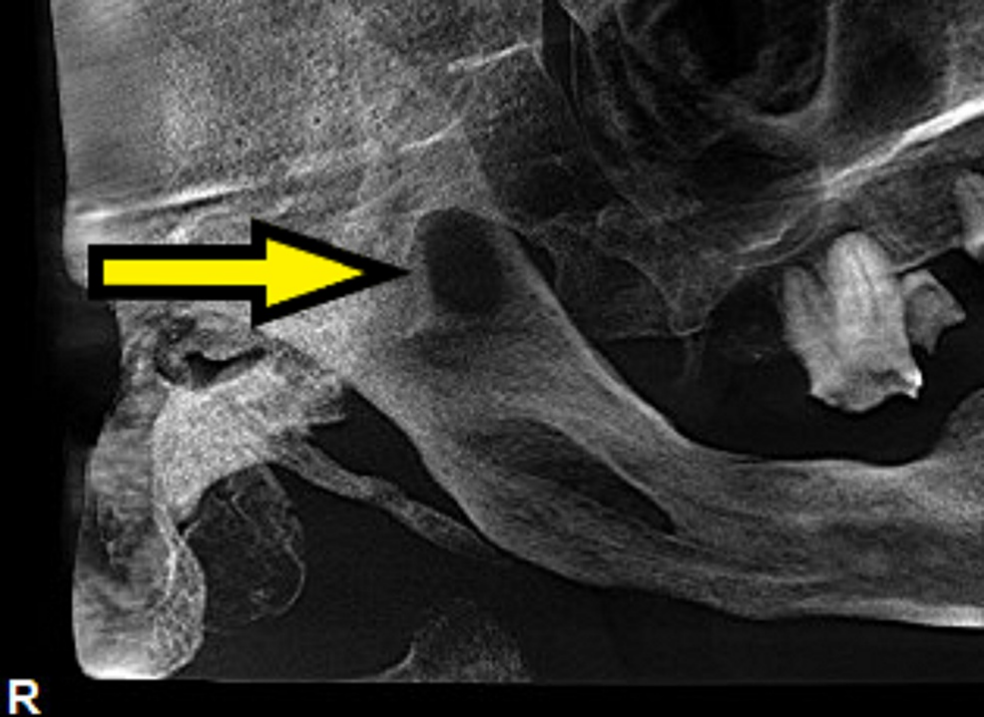
Pre-operative CBCT shows a focal cortical defect opening laterally into the adjacent soft tissue and a sclerotic perifocal mandibular ramus. CBCT, cone beam computed tomography

Cone beam computed tomography (CBCT) showed a focal smooth marginated cortical defect in the right hemi-mandible involving the mandibular notch extending to the base of the adjacent coronoid process. It measured 16 mm x 11 mm (craniocaudal, CC x anteroposterior, AP), and the overlying cortex appears to be thinned out at places. Focal cortical defect opened laterally into the adjacent soft tissue and the peri-focal mandibular ramus appeared sclerotic. The ill-defined soft tissue thickening around the cutaneous defect overlies the right angle of the mandible. The radiographic features along with the clinical features suggested an infective etiology in the right hemi-mandible. MRI was suggested to evaluate for osteomyelitis (Figure 5).

Extraction of 43, 44 and 45, followed by antibiotic therapy with oral ciprofloxacin and ornidazole 500 mg BD, did not resolve the pus discharge and was futile. The surgeon went ahead with the surgical excision of the right coronoid process without performing the MRI because of cost, feasibility, and additional time for the patient.

**Figure 6:**
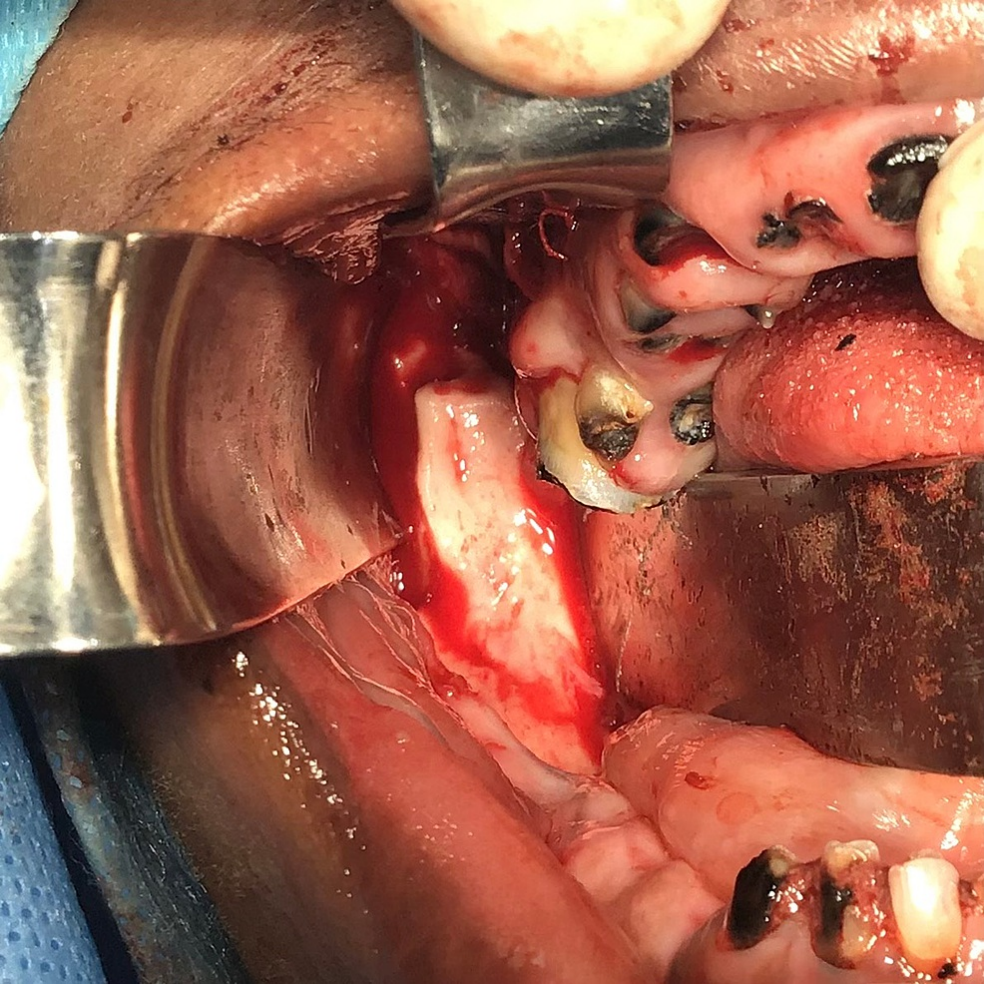
Intra-operative picture showing coronoidotomy of the right side of the mandible.

Anemia was addressed by performing a blood transfusion during the pre-anesthetic evaluation. A right coronoidotomy along with full mouth extraction was performed under general anesthesia and the specimen was sent for histopathological evaluation (Figure 6).

**Figure 7:**
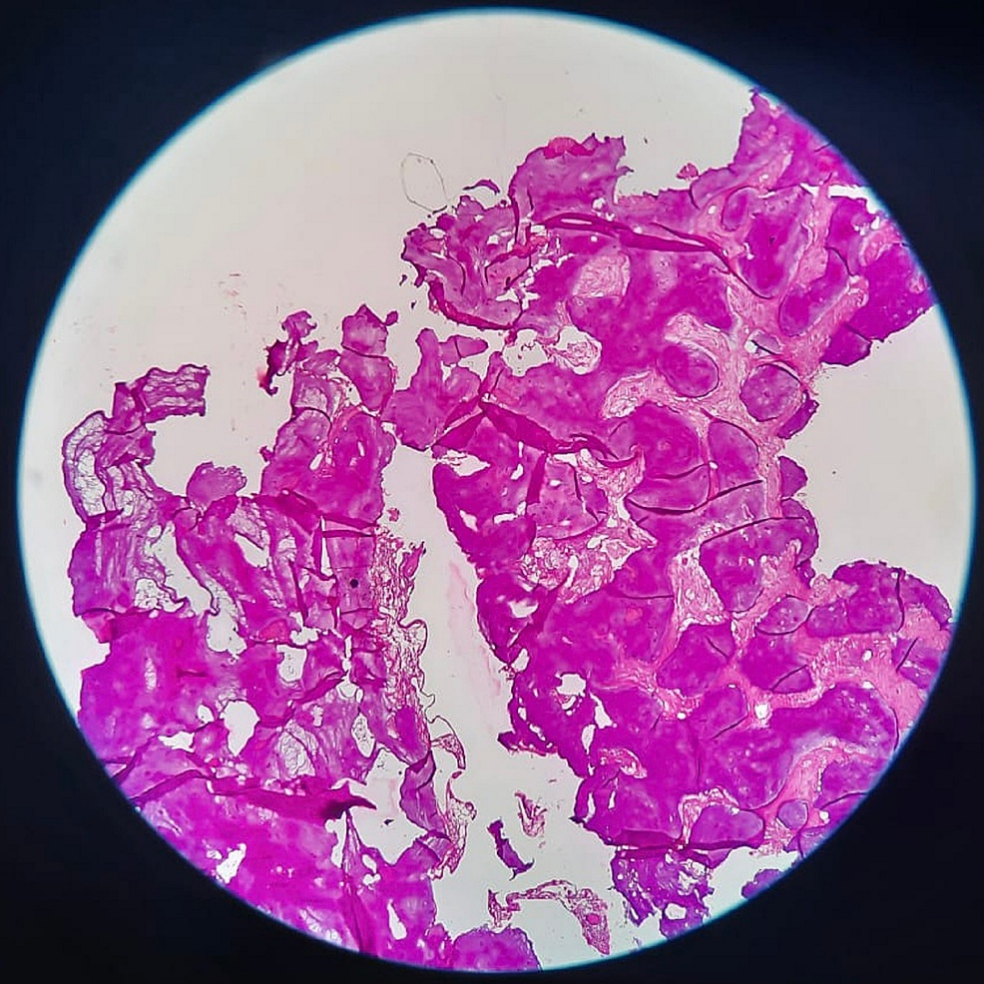
Histopathological slide of decalcified section of bone showing mature trabeculae of lamellar bone with lacunae devoid of osteocytes.

Histopathologic examination showed dense connective tissue stroma with evidence of several foci of chronic inflammatory cell infiltrate along with many endothelial cell proliferation and few Russell bodies. Evidence of an area of epithelial nest suggestive of the juxtaoral organ of Chievitz was observed. The decalcified section of bone showed mature trabeculae of lamellar bone with lacunae devoid of osteocytes along with a few chronic inflammatory cell infiltrates and several areas of hemorrhage (Figure 7).

**Figure 8:**
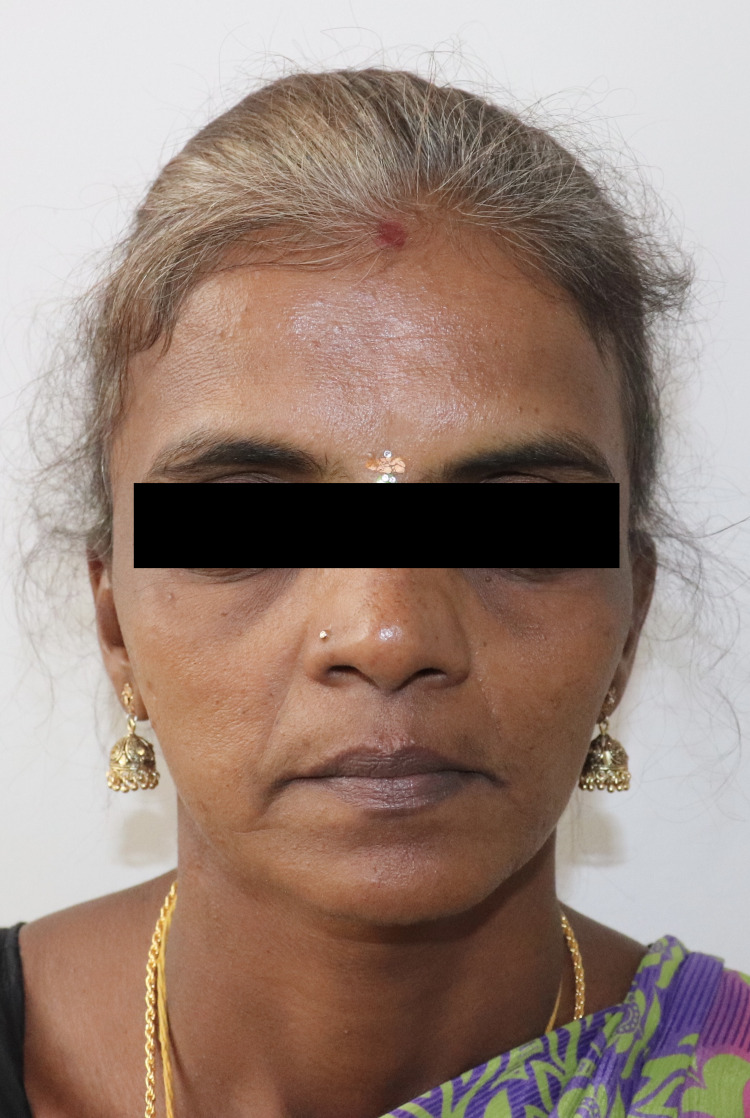
Post-operative extra-oral frontal picture at six-months follow-up.

**Figure 9:**
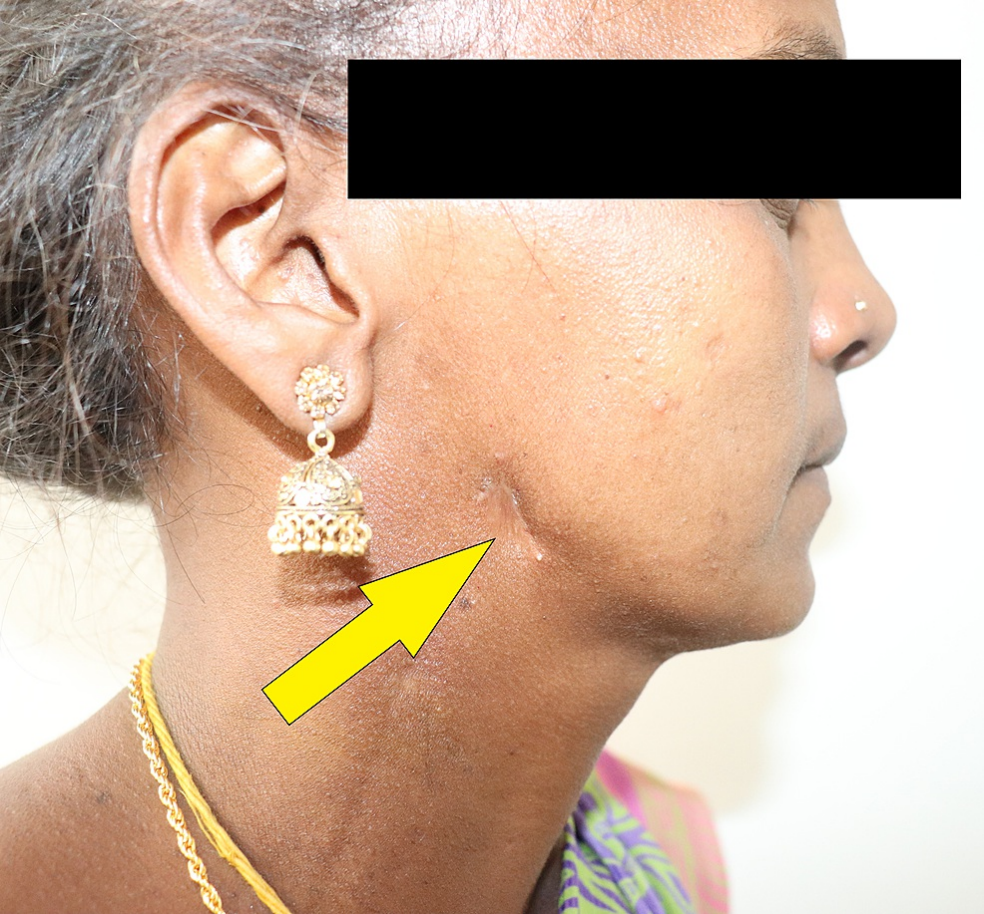
Extra-oral profile picture at six-months follow-up showing obliteration of oro-cutaneous fistula.

The histologic and radiographic patterns were consistent with the chronic type of osteomyelitis. Intravenous cefotaxime 1 g BD, metronidazole 500 mg TID, and paracetamol 1 g BD were administered for three days. She has had regular clinic follow-ups since diagnosis and has noted obliteration of the cutaneous fistula (Figures 8-9).

**Figure 10:**
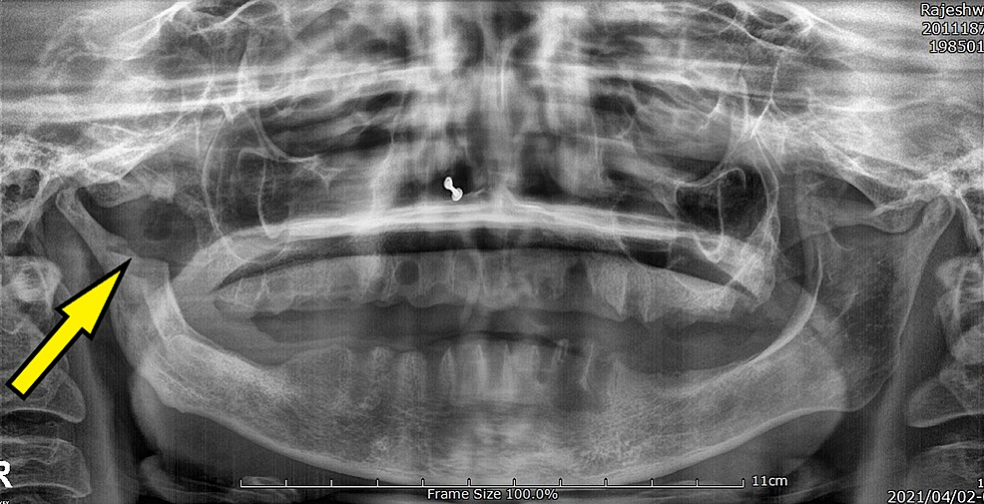
Post-operative OPG at six-months follow-up. OPG, orthopantomograph

Post-surgical OPG reveals resolution of the lesion and is fit to undergo prosthetic rehabilitation of the missing teeth (Figure 10).

## Discussion

Osteomyelitis is a rare, inhomogeneous condition with a varied clinical picture causing potential functional impairment or permanent disability in severe cases. It refers to inflammation of the entire bone, inclusive of the periosteum, cortical and cancellous bone, along with bone marrow. Many classifications exist based on etiology, pathology, anatomical variations, clinical course or radiologic pattern, which makes the comparison between different types hard.

The Zurich classification is recognized as the most reliable classification system which classifies as acute osteomyelitis, secondary chronic osteomyelitis, and primary chronic osteomyelitis [2]. Acute osteomyelitis leads to secondary chronic osteomyelitis and hence is the same disease at a later phase of time.

There are multiple etiologies for osteomyelitis, including inoculum of bacteria, trauma, ischemia, or foreign bodies. Incidence of infection depends on the local immune response of the host, virulence, and load of bacteria, and blood flow to the tissues. In our case, the patient also gave a history of anemia, which is a potential risk factor in the development of osteomyelitis. The bacteria contaminate the bone and proliferate and multiply in the bone marrow. It reaches the periosteum through the Haversian and Volkmann canals forming an edema, which hinders blood flow to the bone and subsequently builds a sequestrum [3-4].

Secondary chronic osteomyelitis is currently considered the most frequent form of osteomyelitis with an incidence of 70% while the incidence rate of acute osteomyelitis is 17% and primary chronic osteomyelitis is 10%. There is a surge in incidence because of the advances in diagnostic imaging techniques. Plain radiography, like panoramic radiograph, is the first-line imaging test in suspected osteomyelitis, as it gives simultaneous information of the maxilla and mandible and aids in identifying and excluding differentials like a fracture. It plays a pivotal role in diagnosing osteomyelitis, although it cannot diagnose the condition independently [5-6].

Radiographically, osteomyelitis is confirmed by the increase in thickness of lamina dura of alveolus, sclerotic variation around the mandibular canal, sclerotic variation of the maxillary bone, and simultaneous destruction and resorption of bone tissue and pattern. But, discovering such signs may be questionable at an early stage of four to eight days after onset of osteomyelitis [4]. Paradoxically, our patient had a well-defined radiolucency mimicking a cyst on the right coronoid process which was completely asymptomatic. Well-defined osteolytic bone lesions have a plethora of differentials at different age groups. Hence, it is necessary to rule out differentials like fibrous dysplasia, eosinophilic granuloma, giant cell tumor, osteoblastoma, aneurysmal bone cyst, solitary bone cyst, hyperparathyroidism (Brown tumor), infection, chondroblastoma, metastasis, and myeloma [7]. Because of ongoing symptoms and high clinical suspicion of the lesion, CBCT was performed. It revealed a focal smooth marginated cortical defect in the right coronoid process of the mandible with a sclerotic appearance. MRI correlation was suggested to evaluate for osteomyelitis.

MRI demonstrates bone marrow edema, confirms abscesses, and delineates extra-osseous disease spread. MRI, being the best imaging modality for establishing the diagnosis, was not performed considering the feasibility and additional cost for the patient [8-9].

The goal of the therapy is to treat the infection with antibiotics and improving the local blood flow by removing the infected bone [3,10]. Treatment aims for sufficient pain management, limiting the spread of infection, prevention of pathological fractures, and the recurrent onset of active infection. Conservative treatment was initiated by extraction of 43, 44, and 45 followed by antibiotic therapy. But, it did not improve the symptoms. Hence, surgical removal of the coronoid process was planned under general anesthesia. A full mouth extraction was done along with right coronoidectomy. The extraoral cutaneous fistula was excised and closure was done in layers. Post-surgically, antibiotics and painkillers were administered and the patient was discharged uneventfully.

Therapeutic success is characterized by a symptom-free state after the intervention. Acute and chronic osteomyelitis have a success rate of approximately 75%. Early treatment, including antibiotic therapy, is necessary to prevent the development of complications like septic arthritis, pathological fractures, squamous cell carcinoma, amyloidosis (rare), bone deformity, systemic infection, and contiguous soft tissue infection [11]. Following surgical debridement, it is essential to closely follow-up with prolonged antibiotics and provides meticulous wound care. The patient transitioned from parenteral antibiotics to oral antibiotics after discharge to complete her antibiotic course. Family members and caregivers also play an integral role in offering adequate care and support. The morbidity of osteomyelitis can be decreased only through an interprofessional team.

## Conclusions

This case shows the utility of the imaging modalities to better diagnose osteomyelitis so that treatment options can be introduced at an earlier stage. In the end, biopsy is considered the gold standard. The early detection of osteomyelitis is critical since treatment options are currently available with the goal of improving the quality of life in affected patients. Current research has shown promise that the aforementioned imaging modalities may also help assess response to therapy. Initially, the lesion was thought to be cyst secondary to odontogenic or nonodontogenic cysts, but the development of imaging, CT scanning revealed focal cortical defect opening laterally into the adjacent soft tissue and a sclerotic perifocal mandibular ramus. Understanding the information got from radiologic studies and correlating them with clinical symptoms is a critical step in diagnosing and which surgical procedure is necessary.
